# Prognostic Biomarkers of Mortality in Older Patients Without Cancer in Home Healthcare

**DOI:** 10.7759/cureus.54326

**Published:** 2024-02-16

**Authors:** Nayuta Shimizu, Syuichi Nakai, Takeshi Takahashi, Masahiro Takihata, Kazuhiko Kotani

**Affiliations:** 1 Division of Community and Family Medicine, Jichi Medical University, Shimotsuke-City, JPN; 2 Department of Medicine, Harmony Clinic, Saitama-City, JPN; 3 Department of Medicine, Miura Central Clinic, Miura-City, JPN

**Keywords:** home care, hemoglobin, aspartate aminotransferase, all-cause mortality, albumin

## Abstract

Introduction

The number of patients without cancer who receive home healthcare is increasing; however, prognostic prediction is challenging among them. This study aimed to investigate the prognostic value of generic biomarkers for mortality in patients without cancer who receive home healthcare.

Materials and methods

The multicenter retrospective cohort study included 114 older patients without cancer, of which 12 (10.5%) died during the study period. The median (interquartile range (IQR)) of the study observation period was 181 (49-293) days. Generic biomarkers included hemoglobin (Hb), albumin (Alb), C-reactive protein (CRP), estimated glomerular filtration rate (eGFR), aspartate aminotransferase (AST), and alanine aminotransferase (ALT). A multivariate-adjusted Cox proportional hazard model on all-cause mortality was used to calculate hazard ratio (HR) and 95% confidence interval (95% CI) for each biomarker. The cut-off values of each biomarker were calculated by receiver operating characteristic curve analysis. The performance of cut-off values was evaluated by time-dependent area under the curves (AUCs).

Results

The median (IQR) of AST was 13 (10-21) U/L. The biomarkers significantly predictive of mortality were Hb (fully adjusted HR: 0.41; 95% Cl: 0.25 - 0.70), Alb (HR: 0.41; 95% Cl: 0.02 - 0.69), and AST (HR: 1.09; 95% Cl: 1.00 - 1.18), along with male sex (HR: 4.07; 95% Cl: 1.15 - 14.35). The AUC of a cut-off value of AST (> 31 U/L) at 360 days was 0.72 (95% CI 0.71 - 0.72; p < 0.01), which outperformed the AUCs for Hb and Alb.

Conclusion

AST, in addition to Hb and Alb, may be useful for predicting the prognosis of older patients without cancer, who had a normal-to-mild increased level of AST, in home healthcare settings. Larger-sample and longer follow-up studies will be warranted.

## Introduction

In developed countries, the demand for home healthcare is increasing with an aging population [1,2]. Moreover, home healthcare is being promoted in Japan, where it is provided to patients who are unable to visit medical institutions on their own, such as older adults who require nursing care [3]. Patients receiving home healthcare are varied and include those with advanced stages of cancer and those without cancer, and the number of non-cancer patients is increasing [4]. Compared to patients with cancer, those without cancer typically have a longer duration of home healthcare with various clinical courses due to a diverse range of non-cancerous diseases, leading to a difficult prognosis and a higher average age of death [3,5-7].

The ability to predict a patient’s prognosis is useful for end-of-life planning for all patients, including those without cancer [6,8]. Almost all studies on prognostic prediction involve patients with cancer only or both with and without cancer [3,9,10]; thus, the studies on patients without cancer are necessary with some objective measures as patient prognosis is one of the main factors contributing to medical decisions in end-of-life planning [11]. Several generic biomarkers are commonly measured in clinical practice in Japan [12]. In community-dwelling older people, generic biomarkers such as hemoglobin (Hb) [13], aspartate aminotransferase (AST) [14], alanine aminotransferase (ALT) [15], estimated glomerular filtration rate (eGFR) [16], C-reactive protein (CRP) [17], and albumin (Alb) [18] were associated with the risk of all-course mortality. Although Hb [9-10] and Alb [3,10] have been previously studied as potential prognostic biomarkers in home healthcare, there is still a lack of research on other routinely employed generic biomarkers. Therefore, our study aimed to investigate the prediction of those generic biomarkers for all-cause mortality in older adults without cancer who received home healthcare.

## Materials and methods

Participants

The multicenter retrospective cohort study was conducted between January 2016 and 2020. Japanese patients were included if they were aged 65 years or more and receiving care from two home healthcare clinics based on the chart review. These patients had common disease conditions. The exclusion criteria were (1) aged less than 65 years; (2) cancer history; and (3) insufficient data. Overall, 114 older patients without cancer were enrolled in this study. The present study conforms to the Declaration of Helsinki and was approved by the Institutional Ethics Committee.

Data

Data on patient age, sex, generic biomarkers (Hb, Alb, CRP, AST, ALT, eGFR), duration of home healthcare, and prognostic information of death were collected from electronic medical records and death certificates.

Hb was measured by the cyanmethemoglobin method. Alb was measured by the modified bromocresol purple method. CRP was measured by the immune assay. AST and ALT were measured by the ultraviolet method. eGFR was calculated from the serum creatinine measured by the enzymatic method.

Statistics

Data for descriptive statistics are presented as the median and interquartile range (IQR) for continuous variables or as a percentage of the population. For survival analysis, hazard ratio (HR) and 95% confidence interval (95% CI) were determined using univariate, sex- and age-adjusted, and fully multivariate-adjusted Cox proportional hazard models on all-cause mortality (the outcome). Moreover, measured variables, such as age, sex, and generic biomarkers, were used in the multivariate analysis. Then, receiver operating characteristic (ROC) curve analysis was performed using all-cause mortality for the biomarkers that significantly predicted mortality in the multivariate Cox proportional hazards model, and the closest point from the upper left corner was used as the cut-off value of each biomarker. Time-dependent ROC curve analysis was used to evaluate the performance of the cut-off values, and the area under the curves (AUCs) and 95% CI were calculated by 1000 bootstraps. The cut-off values were evaluated at different time points: 90-day, 180-day, and 360-day. Statistical analyses were performed using BellCurve for Excel version 3.23 (Social Survey Research Information Co., Ltd., Tokyo, Japan) and R version 4.3.1 (R Foundation for Statistical Computing, Vienna, Austria). A P-value < 0.05 was considered statistically significant.

## Results

Table 1 presents the baseline characteristics of the study patients. The median age (IQR) of participants was 87 (82-91) years, and 24.6% were male. The median (IQR) of the study observation period was 181 (49-293) days. The median (IQR) of AST was 13 (10-21) U/L (the min-max level was 6-77 U/L).

**Table 1:** Baseline clinical characteristics of patients Note: Data are shown as the median (interquartile range). Alb, albumin; AST, aspartate aminotransferase; ALT, alanine aminotransferase; CRP, C-reactive protein; eGFR, estimated glomerular filtration rate; Hb, hemoglobin

Characteristics	% or median (n = 114)
Male (%)	24.6
Age (years)	87 (82 - 91)
Hb (g/dL)	11.8 (10.4 - 12.8)
Alb (g/dL)	3.5 (3.2 - 3.8)
eGFR (mL/min/1.73m^2^)	65 (44 - 77)
AST (U/L)	21 (17 - 26)
ALT (U/L)	13 (10 - 21)

Of the 114 patients, 12 (10.5%) died during the period. The cause of death was attributed to disease conditions such as cognitive disorders, hypertension, renal failure, and heart failure. As shown in Table 2, the biomarkers that significantly predicted mortality were Hb (fully multivariate-adjusted HR: 0.41; 95% Cl: 0.25 - 0.70; p < 0.01), Alb (HR: 0.41; 95% Cl: 0.02 - 0.69; p < 0.05), AST (HR: 1.09; 95% Cl: 1.00 - 1.18; p < 0.05), and male sex (HR: 4.07; 95% Cl: 1.15 - 14.35; p < 0.05). The impact of respective biomarkers on mortality seemed to be consistent when analyzing the univariate and adjusted HR.

**Table 2:** Prediction of all-cause mortality Alb, albumin; AST, aspartate aminotransferase; ALT, alanine aminotransferase; CI, confidence interval; CRP, C-reactive protein; eGFR, estimated glomerular filtration rate; Hb, hemoglobin; HR, hazard ratio

	Univariate analysis		Sex- and age-adjusted analysis		Multivariate analysis	
Characteristics	HR (95% Cl)	P-value	HR (95% Cl)	P-value	HR (95% Cl)	P-value
Male sex	5.21 (1.65 - 16.48)	< 0.01	-	-	4.07 (1.15 - 14.35)	< 0.05
Age (years)	1.00 (0.92 - 1.09)	0.98	-	-	0.90 (0.78 - 1.04)	0.14
Hb (g/dL)	0.51 (0.36 - 0.71)	< 0.01	0.53 (0.37 - 0.76)	< 0.01	0.41 (0.25 - 0.70)	< 0.01
Alb (g/dL)	0.17 (0.07 - 0.43)	< 0.01	0.12 (0.03 - 0.49)	< 0.01	0.41 (0.02 - 0.69)	< 0.05
eGFR (mL/min/1.73m^2^)	0.99 (0.97 - 1.01)	0.24	0.99 (0.96 - 1.01)	0.2	0.99 (0.97 - 1.02)	0.52
AST (U/L)	1.04 (1.01 - 1.07)	< 0.01	1.04 (1.01 - 1.07)	< 0.05	1.09 (1.00 - 1.18)	< 0.05
ALT (U/L)	1.04 (1.00 - 1.07)	< 0.01	1.03 (1.00 - 1.07)	0.09	0.98 (0.90 - 1.08)	0.71
CRP (mg/dL)	1.14 (0.98 - 1.32)	0.09	1.07 (0.91 - 1.25)	0.42	0.95 (0.76 - 1.19)	0.67

The cut-off values of Hb, Alb, and AST were 11.1 g/dL, 3.3 g/dL, and 31 U/L, respectively. The number of patients with Hb < 11.1 g/dL, Alb < 3.3 g/dL, and AST > 31 U/L were 41 (36.0%), 31 (27.2%), and 18 (15.8%), respectively. The cut-off values that significantly predicted mortality were Alb < 3.3 g/dL (fully multivariate-adjusted HR: 5.27; 95% Cl: 1.38 - 20.11; p < 0.05) and AST > 31 U/L (HR: 10.77; 95% Cl: 2.97 - 29.03; p < 0.01). As shown in Table 3, the AUCs of AST > 31 were 0.67 (95% CI 0.66 - 0.67; p < 0.01), 0.68 (95% CI 0.67 - 0.69; p < 0.01), and 0.72 (95% CI 0.71 - 0.72; p < 0.01) at 90, 180 and 360 days, respectively. The AUC at 360 days for Hb was 0.67 (95% Cl: 0.66 - 0.67; p < 0.01) and that for Alb was 0.68 (95% Cl: 0.68 - 0.69; p < 0.01).

**Table 3:** The time-dependent AUC of cut-off values of each biomarker Alb, albumin; AST, aspartate aminotransferase; AUC, area under the curve; CI, confidence interval; Hb, hemoglobin

	90-days		180-days		360-days	
Characteristics	AUC (95% Cl)	P-value	AUC (95% Cl)	P-value	AUC (95% Cl)	P-value
Hb < 11.1 g/dL	0.74 (0.73 - 0.74)	< 0.01	0.71 (0.71 - 0.72)	< 0.01	0.67 (0.66 - 0.67)	< 0.01
Alb < 3.3 g/dL	0.73 (0.73 - 0.74)	< 0.01	0.72 (0.72 - 0.73)	< 0.01	0.68 (0.68 - 0.69)	< 0.01
AST >31 U/L	0.67 (0.66 - 0.67)	< 0.01	0.68 (0.68 - 0.69)	< 0.01	0.72 (0.71 - 0.72)	< 0.01

## Discussion

This is the first description to indicate that AST, in addition to Hb and Alb, is an independent prognostic biomarker for older patients without cancer, who had a normal-to-mild increased level of AST, in the home healthcare settings. These findings suggest that the AST level could be measured at baseline and utilized in combination with the helpful biomarkers (Alb and Hb) as reported previously [3,9-10] for estimating prognosis in older patients. The time-dependent AUC of AST > 31 also increased by 360 days (Table 3). Furthermore, because the upper limit of the AST level reference range is 30 U/L [19], the cut-off value of AST > 31 U/L would be easily acceptable and usable for care workers and general people when applying AST to home healthcare settings.

In clinical practice, AST and ALT levels are typically used as an indicator of liver function [20] and their increases indicate a poor prognosis in the general older population [14-15,21-22]. Moreover, the AST level is reported to have a better predictive ability for all-cause mortality than the ALT level [14]. Notably, AST is known as a less specific marker of liver function than ALT; the AST level reflects the damage to other tissues such as those of the heart, skeletal muscle, and kidneys [14]. The causes of death in the home healthcare settings are not commonly liver-specific diseases but rather multiorganic disorders; thus, the prognostic value of AST might be explained by such complex tissue damage in older patients without cancer [14,20].

In the present study, we observed that low levels of Hb or Alb were associated with a poor prognosis in patients in home healthcare, which is in line with previous studies [3,9,18]. Low Alb levels are associated with malnutrition [18], undernutrition [23], and inflammation in non-cancerous diseases [24]. Low Hb levels are associated with anemia caused by iron deficiency, chronic renal failure, and inflammation [25]. Thus, the underlying status, as often seen in older patients, could explain the results of the present study.

It is of interest to note the increase in the time-dependent AUC of AST > 31 by 360 days in comparison to that of Hb and Alb. This may indicate the AST level to be useful for the relatively long-term prognostic ability while Hb and Alb are predictive of shorter-term prognosis. Patients without cancer, as studied in the present study, often have a longer duration of home healthcare [3,5-7], which might partly lead to the highlight of the relevance of AST. Since the number of patients without cancer is increasing [4], we expect more studies to find out the clinical relevance of their combinations with more biomarkers such as AST besides Hb and Alb.

Our study had several limitations. First, even though the result was explainable, that was obtained in a comparatively small sample-sized study. Second, although this was a cohort study, a retrospective design was used with a comparatively short observation period. Third, although the study patients were not specific and severe but stable and common disease conditions, the detailed data on diseases and prescribed drugs were unavailable. In older people, multimorbidity complications and polypharmacy are usually seen [26]. Polypharmacy may affect liver and renal function [27]. Caution should thus be exercised when generalizing the findings of this study. Larger-sample and longer follow-up studies with detailed data are required in future work.

## Conclusions

This study showed that the AST level, in addition to Hb and Alb, could be useful for predicting the prognosis of older patients without cancer, who had a normal-to-mild increased level of AST, in home healthcare settings. High levels of AST might also predict a relatively long-term poor prognosis in comparison to low levels of Hb and Alb. We expect to find out the relevance of combinations of those biomarkers on clinical courses in such patients. Further studies are warranted to accumulate the findings.

## References

[1] Tsutsui T (2014). Implementation process and challenges for the community-based integrated care system in Japan. Int J Integr Care.

[2] Shimizu N, Kotani K (2022). Health information exchange in relation to point-of-care testing in home care: issues in Japan. Clin Chim Acta.

[3] Kaneko M, Watanabe T, Fujinuma Y, Yokobayashi K, Matsushima M (2021). Overall mortality in older people receiving physician-led home visits: a multicentre prospective study in Japan. Fam Pract.

[4] Kosaka M, Miyatake H, Arita S, Masunaga H, Ozaki A, Nishikawa Y, Beniya H (2020). Emergency transfers of home care patients in Fukui Prefecture, Japan. A retrospective observational study. Medicine (Baltimore).

[5] Hirahara S (2004). The present state and subject of home hospice for patients with non-cancer disorders [Article in Japanese]. Gan To Kagaku Ryoho.

[6] Boyd M, Frey R, Balmer D (2019). End of life care for long-term care residents with dementia, chronic illness and cancer: prospective staff survey. BMC Geriatr.

[7] Masumoto S, Sato M, Ichinohe Y, Maeno T (2019). Factors facilitating home death in non-cancer older patients receiving home medical care. Geriatr Gerontol Int.

[8] Jones NR, Roalfe AK, Adoki I, Hobbs FD, Taylor CJ (2019). Survival of patients with chronic heart failure in the community: a systematic review and meta-analysis. Eur J Heart Fail.

[9] Todoroki K, Ikeya Y, Fukui S (2017). The vital prognosis of elderly adults living in a group home in their mid-eighties. J Physiol Sci.

[10] Kitamura K, Nakamura K, Nishiwaki T, Ueno K, Hasegawa M (2010). Low body mass index and low serum albumin are predictive factors for short-term mortality in elderly Japanese requiring home care. Tohoku J Exp Med.

[11] Arruda LM, Abreu KP, Santana LB, Sales MV (2020). Variables that influence the medical decision regarding Advance Directives and their impact on end-of-life care. Einstein (Sao Paulo).

[12] Ichihara K, Itoh Y, Lam CW (2008). Sources of variation of commonly measured serum analytes in 6 Asian cities and consideration of common reference intervals. Clin Chem.

[13] Karoopongse E, Srinonprasert V, Chalermsri C, Aekplakorn W (2022). Prevalence of anemia and association with mortality in community-dwelling elderly in Thailand. Sci Rep.

[14] Xie K, Chen CH, Tsai SP (2019). Loss of life expectancy by 10 years or more from elevated aspartate aminotransferase: finding aspartate aminotransferase a better mortality predictor for all-cause and liver-related than alanine aminotransferase. Am J Gastroenterol.

[15] Kawamoto R, Kikuchi A, Akase T, Ninomiya D, Tokumoto Y, Kumagi T (2022). Association between alanine aminotransferase and all-cause mortality rate: findings from a study on Japanese community-dwelling individuals. J Clin Lab Anal.

[16] Oh SW, Kim S, Na KY, Kim KW, Chae DW, Chin HJ (2014). Glomerular filtration rate and proteinuria: association with mortality and renal progression in a prospective cohort of a community-based elderly population. PLoS One.

[17] Giovannini S, Onder G, Liperoti R (2011). Interleukin-6, C-reactive protein, and tumor necrosis factor-alpha as predictors of mortality in frail, community-living elderly individuals. J Am Geriatr Soc.

[18] Cabrerizo S, Cuadras D, Gomez-Busto F, Artaza-Artabe I, Marín-Ciancas F, Malafarina V (2015). Serum albumin and health in older people: review and meta analysis. Maturitas.

[19] Ichihara K, Yomamoto Y, Hotta T (2016). Collaborative derivation of reference intervals for major clinical laboratory tests in Japan. Ann Clin Biochem.

[20] Suciu A, Abenavoli L, Pellicano R, Luzza F, Dumitrascu DL (2020). Transaminases: oldies but goldies. A narrative review. Minerva Gastroenterol Dietol.

[21] Ke P, Zhong L, Peng W (2022). Association of the serum transaminase with mortality among the US elderly population. J Gastroenterol Hepatol.

[22] Nakajima K, Yuno M, Tanaka K, Nakamura T (2022). High aspartate aminotransferase/alanine aminotransferase ratio may be associated with all-cause mortality in the elderly: a retrospective cohort study using artificial intelligence and conventional analysis. Healthcare (Basel).

[23] Seidu S, Kunutsor SK, Khunti K (2020). Serum albumin, cardiometabolic and other adverse outcomes: systematic review and meta-analyses of 48 published observational cohort studies involving 1,492,237 participants. Scand Cardiovasc J.

[24] Hedlund J (1995). Community-acquired pneumonia requiring hospitalisation. Factors of importance for the short-and long term prognosis. Scand J Infect Dis Suppl.

[25] Goodnough LT, Schrier SL (2014). Evaluation and management of anemia in the elderly. Am J Hematol.

[26] Sönnichsen A, Trampisch US, Rieckert A (2016). Polypharmacy in chronic diseases-Reduction of Inappropriate Medication and Adverse drug events in older populations by electronic Decision Support (PRIMA-eDS): study protocol for a randomized controlled trial. Trials.

[27] Abe J, Umetsu R, Uranishi H (2017). Analysis of polypharmacy effects in older patients using Japanese Adverse Drug Event Report database. PLoS One.

